# Leukocyte telomere length change in children with obesity in the context of an isocaloric fructose restriction intervention

**DOI:** 10.1186/s13098-025-01611-0

**Published:** 2025-03-22

**Authors:** Janet M. Wojcicki, Elissa Epel, Jue Lin, Viva Tai, Jean-Marc Schwarz, Susan M. Noworolski, Ayca Erkin-Cakmak, Kathleen Mulligan, Alejandro Gugliucci, Rob H. Lustig

**Affiliations:** 1 Division of Pediatric Gastroenterology, Hepatology and Nutrition, University of California, San Francisco, 550 16th Street, 4th Floor, San Francisco, CA 94134-0136 USA; 2 Department of Psychiatry, University of California, San Francisco, San Francisco, CA USA; 3 Department of Medicine, University of California, San Francisco, USA; 4 Basic Sciences, College of Osteopathic Medicine, Touro University-California, Vallejo, CA USA; 5 Department of Radiology and Biomedical Imaging, University of California, San Francisco, San Francisco, CA USA; 6 Department of Pediatrics, University of California, San Francisco, San Francisco, CA USA; 7 Department of Research, College of Osteopathic Medicine, Touro University-California, Vallejo, CA USA; 8 Department of Biochemistry and Biophysics, University of California, San Francisco, USA

## Abstract

**Background:**

Few studies have evaluated changes in leukocyte telomere length (LTL) over a short time period (e.g. 1 week). LTL shortening is accelerated by exposure to inflammation and reactive oxygen species (ROS) damage.

**Methods:**

In the context of an isocaloric fructose restriction study that was conducted with 43 Black and Latinx children over a 9-day period, we evaluated the relationship between metabolic health at baseline and metabolic changes and LTL at baseline and %LTL change over the follow-up period. Linear regression models were used to assess associations between metabolic correlates and LTL at baseline and LTL changes over 9 days.

**Results:**

Overall children lost − 0.05 ± 0.14 T/S units or − 2.98 ± 8.74% total change over the follow-up period. Higher concentrations of HDL-C, APO-AI and a greater % of large HDL-C at baseline were associated with reduced LTL attrition rates at day 10 (p < 0.01; p < 0.01 and p = 0.02 respectively). Increases in APO-AI over the follow-up period were associated with increased LTL attrition over the follow-up period (p = 0.03).

**Conclusions:**

In this short term isocaloric fructose restriction study, LTL at baseline and changes in LTL over 9 days were associated with HDL-C and APO-AI and not with any other non-HDL-C lipids. Additional, larger studies are necessary to better understand the interplay between short term fructose restriction, LTL changes and HDL-C/APO-AI.

## Background

Telomeres, the repetitive non-coding DNA sequences at the ends of chromosomes, help prevent DNA damage and degradation as the protective cap of the chromosome. Telomere shortening occurs with each cell division eventually resulting in cellular senescence or apoptosis with the enzyme telomerase, adding 'TTAGGG' repeats to the ends of chromosomes to help maintain overall telomere length and integrity [12]. Shorter telomeres have been associated with incident chronic disease in adults including diabetes mellitus [31] and cardiovascular disease [27] as telomere attrition is not only increased through exposure to inflammation and reactive oxygen species damage but also is thought to be pro-inflammatory with increased release of inflammatory cytokines [15].

Few studies have evaluated short-term leukocyte telomere length (LTL) change over a week or month period. Studies have found acute situations such as sepsis can result in accelerated short term loss of LTL over a 7-day period [21] possibly due to the inflammatory process and oxidative damage associated with sepsis. A large study with a heterogenous sample of intensive care unit (ICU) patients including a subset with sepsis similarly found primarily LTL shortening for sepsis patients but no change or lengthening in some of the other hospitalized ICU patients suggesting a complex interplay between acute disease states and LTL homeostasis [32].

Other studies have suggested the possibility of LTL lengthening in the context of reduced inflammation associated with short term behavioral interventions including an association between meditation or kindness behaviors and LTL lengthening over a three-week time period [7, 9] and strength and exercise training associated with LTL lengthening over an 8-week period in post-menopausal women with obesity [3].

We evaluated changes in LTL in the context of a 9-day fructose restriction study with Latinx and African-American children with obesity associated with overall improvements in metabolic health [17]. Short term studies of LTL changes can provide insight into the possible role of interventions on cellular health prior to discernable changes in anthropometrics or other metabolic indices as our previous studies have demonstrated [22].

## Methods

This follow-up study was conducted as a secondary analysis of the Metabolic Impact of Sucrose Restriction in Obese Children Study (the SUCRE study) designed to evaluate the impact of sugar restriction on children with obesity and co-morbid features of the metabolic syndrome. This longitudinal controlled study enrolled 45 Latinx and African-American children (9–18 years old) at University of California, San Francisco (UCSF) Medical Center and substituted sugar and fructose with complex carbohydrates over a 9-day period as previously well-described [8, 17, 11]. Out of the 45 initially enrolled, 43 (95.6%), completed the longitudinal cohort intervention study [17]. Families were provided with detailed instructions on how to collect weight and maintain dietary records as well as a sent home with food for the 9-day follow-up period. Detailed follow-up support was provided by phone, email and text. Dietary sugar and fructose consumption were reduced to 10% and 4% of caloric intake, respectively. In short, findings from the SUCRE study found overall decreases in diastolic blood pressure, lactate, triglycerides, low density lipoprotein (LDL) and weight [17].

For this follow-up secondary analysis, we assessed metabolic correlates at baseline and change in metabolic markers over the 9-day period in relation to LTL and % change in LTL (defined as changes in LTL over the follow-up period in relation to baseline LTL). Fasting blood draws were conducted on days 0 and 9 and specimens were processed and frozen for batch analysis. Serum insulin concentrations were measured by chemiluminescence on a Siemens Immulite 2000 XPI platform, fasting lipids on a Beckman DXC-600 by blanked timed endpoint, and high-density lipoprotein cholesterol (HDL-C) by homogeneous immunoinhibition (Trinity Biotech) at Pennington Biomedical Research Center (Baton Rouge, LA, USA).

To assess LTL in whole blood drawn on days 0 and 9, the following protocol was followed at the UCSF Blackburn Laboratory. Genomic DNA was extracted from whole blood with QIAamp DNA blood mini extraction kit (QIAGEN cat # 51106) and eluted in 50 ul AE buffer. DNA was quantified by OD260/OD280 and the DNA quality control criteria were OD260/OD280 between 1.7-2.0 and concentration greater than 10ng/μl. All baseline and follow-up samples were measured on the same assay plate. The assay coefficient of variation (CV) was 3.5 +/- 3.5%. The details of telomere length method have been described previously described [4, 16] and can be found on the Telomere Research Network’s website (https://trn.tulane.edu/wp-content/uploads/sites/445/2021/07/Lin-qPCR-protocol-01072020.pdf).  To asses telomerase activity, the following protocol was followed. 10 ml of peripheral blood was collected in BD Vacutainer® CPT tubes with density gradient polymer gel and sodium citrate additives. The PBMC fraction was isolated from each blood sample using density gradient centrifugation according to instructions for CPT tubes. Immediately following centrifugation, the PBMC layer was collected. Cells were washed 3 times in phosphate-buffered saline (PBS) and were re-suspended in PBS and live cells were counted with Trypan blue staining solution with a hemocytometer. Extracts corresponding to 5000 cells/ul were made, based on the protocol provided in the TRAPeze telomerase detection kit (Chemicon, Temecula, CA). The extracts were stored at -80oC until use. Quantification of telomerase activity was measured from the extract using the telomeric repeat amplification protocol (TRAP) as previously described with a commercial kit (TRAPeze®, Chemicon, Temecula, CA). The 293T cancer cell line was used as a positive telomerase activity control and reference standard and telomerase activity was defined as 1 unit = the amount of product from one 293T cell/10000 PBMCs [16, 14].

### Statistical analysis

We used tests of normality including Shapiro–Wilk to evaluate LTL at day 0 and percent LTL change over the 9-day period finding that LTL was normally distributed. As such, we used parametric tests of association including Pearson’s correlation coefficient to assess associations between LTL at day 0 and percent LTL change with continuous predictors. We used t-tests to assess associations between LTL at day 0 and percent LTL change with dichotomous predictors.

Previous studies have indicated that age, sex and racial background are associated with LTL [10, 20, 23]. We did not find associations between age, sex or racial background and LTL at day 0 or % LTL change. As such, we present unadjusted analyses due to the risk of over-adjustment in our small sample size [13].

## Results

Overall the group (n = 43) lost LTL ((−)0.05 ± 0.14 T/S ratio or (−)2.98 ± 8.74% change) and telomerase change of (−)1.04 ± 8.41 over the course of the 9-day fructose restriction. Mean age of the group was 13.3 ± 2.7 years, 62.8% were female and mean BMI z-score was 2.4 ± 0.3 as previously reported [17]. Additional anthropometrics on the participants included a mean weight at baseline of 93.0 ± 22.1 (kg) and a body mass index of 35.6 ± 64 (kg/m^2^) [17].

LTL at baseline was not associated with sex, racial/ethnic background or age (Table 1). Longer LTL trended towards significance with greater large HDL% (Coeff = 0.008(0.004); p = 0.05) as did LTL with the ratio of intermediate and large HDL% to small HDL % (Coeff = 0.009 (0.005); p = 0.10). Baseline insulin levels and HOMA-IR were inversely associated with LTL length at baseline Coeff = (−)0.004(0.002); p = 0.03) and Coeff = (−)0.0009 (0.004); p = 0.03) (Table 1).

**Table 1:** Metabolic and demographic variables at baseline in relation LTL and changes in LTL

LTL	LTL at baseline T/S ratio (n = 43)	Percent change in LTL (T/S ratio) (n = 43)
	Coeff (SE) (p value)	Coeff (SE) (p value)
Variables at baseline		
Demographics		
Sex (Female)	0.065(0.073) (0.38)	4.472 (2.722) (0.11)
Age, years	− 0.014(0.017) (0.42)	0.198 (0.675) (0.77)
Race/Ethnicity (Latinx vs African-American)	− 0.104 (0.072) (0.27)	− 2.779 (2.778) (0.32)
Anthropometrics		
Weight, kg	− 0.002(0.002) (0.13)	− 0.007 (0.062) (0.91)
Lipids		
HDL-Cholesterol, mg/dL	0.003(0.004) (0.48)	**0.387 (0.134) (< 0.01)**
LDL-Cholesterol, mg/dL	0.001 (0.001) (0.34)	0.072(0.055) (0.20)
APO-AI, mg/dL	0.009 (0.05) (0.86)	**5.821 (1.979) (< 0.01)**
APO-B, mg/dL	0.05 (0.43) (0.91)	− 17.701 (18.324) (0.34)
Total Cholesterol, mg/dL	0.0008 (0.001) (0.49)	0.061 (0.044) (0.17)
Triglycerides, mg/dL	− 0.0002(0.005) 0.66	− 0.009 (0.018) 0.64
Intermediate-Large HDL %*	0.003 (0.006) 0.18	0.471 (0.242) 0.06
Large HDL %*	0.008(0.004) 0.05	**0.416 (0.175) 0.02**
Small HDL %*	− 0.008(0.006) (0.15)	− 0.444 (0.236) (0.07)
Intl-Large HDL%/ Small HDL%*	0.009(0.005)( 0.10)	0.261 (0.227) (0.26)
HDL/TL ratio	0.124 (0.101) (0.23)	3.273 (3.869) (0.40)
Metabolic		
Glucose, mg/dL	− 0.002(0.004) (0.64)	0.046(0.166) (0.78)
Insulin, uU/mL	− **0.004 (0.002) (0.03)**	− 0.075 (0.071) (0.30)
HOMA-IR	− **0.0009 (0.004) (0.03)**	− 0.015 (0.016) (0.35)
Other biomarkers		
Telomerase	0.0003(0.004) (0.95)	0.004 (0.157) (0.98)

Bold values indicate statistical significance,* p* < 0.05

^*^According to manufacturer’s reported as % of total HDL lipid AUC

Percentage change in LTL from baseline to day 9 was also associated with higher HDL-C, APO-AI and the % of large HDL-C at baseline (Coeff = 0.387 (0.134); p < 0.01; Coeff = 5.821 (1.979); p < 0.01); Coeff = 0.416 (0.175); p = 0.02). The percentage of greater number of intermediate and large HDL-C at baseline trended towards greater significance with % change in LTL from baseline to follow-up (Coeff = 0.471 (0.242); p = 0.06) and small % HDL-C at baseline trended towards an inverse association with greater % LTL change (Coeff = (−)0.444(0.236); p = 0.07) (Table 1).

We did not find any associations between change in metabolic parameters or anthropometrics during the follow-up period and LTL at baseline. However, changes in APO-AI were inversely associated with percentage changes in LTL (Coeff = − 4.212 (1.802); p = 0.03) and higher HDL-C trended towards inverse association with smaller percentage change in LTL (Coeff = − 0.530 (0.287); p = 0.07) (Table 2).

**Table 2:** Metabolic changes in relationship to LTL and baseline and percent change in LTL

	LTL at baseline (n = 43)	Percent change in LTL over follow-up period (n = 43)
	Coeff (SE) (p value)	Coeff (SE) (p value)
Changes in variable over follow-up period		
Lipids		
HDL-Cholesterol, (mg/dL)	− 0.006 (0.008) (0.93)	− 0.530 (0.287) (0.07)
LDL-Cholesterol (mg/dL)	− 0.002 (0.002) (0.37)	− 0.053 (0.089) (0.56)
APO-AI (mg/dL)	− 0.004 (0.042) (0.93)	− **4.212 (1.802) (0.03)**
APO-B (mg/dL)	− 0.029 (0.799) (0.65)	20.978 (34.370) (0.55)
Triglycerides (mg/dL)	0.00009 (0.0006) (0.88)	− 0.003 (0.022) (0.87)
Total Cholesterol (mg/dL)	− 0.0009 (0.002) (0.63)	− 0.062 (0.071) (0.38)
Intermediate-Large HDL%*	− 0.008 (0.008) (0.34)	− 0.249 (0.354) (0.49)
Small HDL-Cholesterol%*	0.0096 (0.008) (0.23)	0.220 (0.394) (0.84)
Large HDL-Cholesterol%*	− 0.002 (0.005) (0.80)	− 0.020 (0.23) (0.93)
Intl-Large HDL-Cholesterol%*		
/Small HDL-Cholesterol%*	− 0.019 (0.01) (0.18)	− 0.072 (0.606) (0.91**)**
HDL/TL ratio	0.045 (0.14) (0.75)	4.913 (5.321) (0.36)
Metabolic		
Glucose (mg/dL)	0.011 (0.008) (0.18)	− 0.072 (0.332) (0.83)
Insulin (uU/mL)	0.003 (0.002) (0.18)	0.018 (0.097) (0.85)
HOMA-IR	0.0008 (0.0006) (0.14)	0.003 (0.022) (0.90)
Anthropometrics		
Weight (kg)	0.026 (0.032) (0.42)	− 1.250 (1.233) (0.32)
Other Biomarkers		
Telomerase (per 10,000 cells)	0.00099 (0.004) (0.82)	0.157 (0.157) (0.34)

Bold values indicate statistical significance,* p* < 0.05

^*^According to manufacturer’s reported as % of total HDL lipid AUC

## Discussion

This short-term fructose isocaloric restriction study had an overall LTL loss over 9 days in our study population of Latinx and Black children with obesity. LTL naturally declines as human age or are exposed to oxidative stress; however, the natural history of LTL changes over short time periods have rarely been assessed [5]. Previous studies have not evaluated short-term LTL changes in children with obesity in relation to dietary changes. Lipids, in particular HDL-C and APO-AI were associated with LTL changes in this study.

Our findings of associations between HDL-C and APO-AI and LTL lengthening or diminished shortening is similar to other findings in adults that have emphasized the anti-oxidant and anti-inflammatory role that HDL-C plays that could positively impact LTL [6].

We did find a paradoxical inverse association between % change in LTL over the follow-up period and APO-AI. Similarly, HDL-C changes trended towards significance, also in a similar direction in association with % change in LTL. Our study team had a similar finding with changes in % LTL associated inversely with APO-AI changes over a 10 months sugar sweetened beverage sales ban in adults with obesity [26]. Although a meta-analysis of 24 trials indicated that fructose exerted no effect on HDL-C [30], a recent study found significant racial differences between Black and White adults in the role of HDL-C in coronary heart disease (CHD) risk in adults with no associations between low or high HDL-C and incident CHD for Black adults. As our study was compromised only of Black and Latinx youth, changes in HDL-C in this population group likely do not reflect other published findings from White adults [29].

LTL is shorter in neutrophils than lymphocytes; we did not asses leukocyte composition in our analyses and increases or decreases in LTL over time could reflect changes in leukocyte composition [24]. Studies in obese children, however, have not found any changes in neutrophil to lymphocyte ratio in the context of metabolic disease severity [19] or in relation to changes in BMI status [28]. Meanwhile, previous studies have found a negative association between neutrophil count and HDL-C but these studies were with adults [18, 25]. As HDL-C and APO-AI are fundamental to the immune system response and their association with LTL changes may differ from non- HDL-C lipids [2], our paradoxical findings suggest the need for further exploration of the impact of fructose restriction on immune system response among children with obesity. For example, previous studies have indicated that the HDL/APOA-I system can turn pro-inflammatory in the context of cholesterol depletion [2].

While ours is the first study to assess short term LTL changes in the context of fructose restriction in children with obesity, additional longitudinal studies are needed to confirm our findings as well as other studies with different population groups. Other short-term LTL studies have found that accelerated attrition may happen in the context of hospitalization and the stress of painful hospital procedures for preterm infants but few other short-term LTL studies have been conducted including none with obese adolescents [1]. Additionally, future studies should collect more detailed markers of immune system response to better understand the interplay between LTL, immune system changes and fructose restriction.

## Data Availability

Data is available on request to the corresponding author.
